# Supplementary material for: An intravital window to image the colon in real time
Source: Nat Commun. 2019 Dec 11;10:5647. doi: 10.1038/s41467-019-13699-w (PMC6906443; doi:10.1038/s41467-019-13699-w)
Supplement: Supplementary file 1 — Supplementary Information [file 41467_2019_13699_MOESM1_ESM.pdf]

Supplementary Information

An intravital window to image the colon in real time

Rakhilin et al.

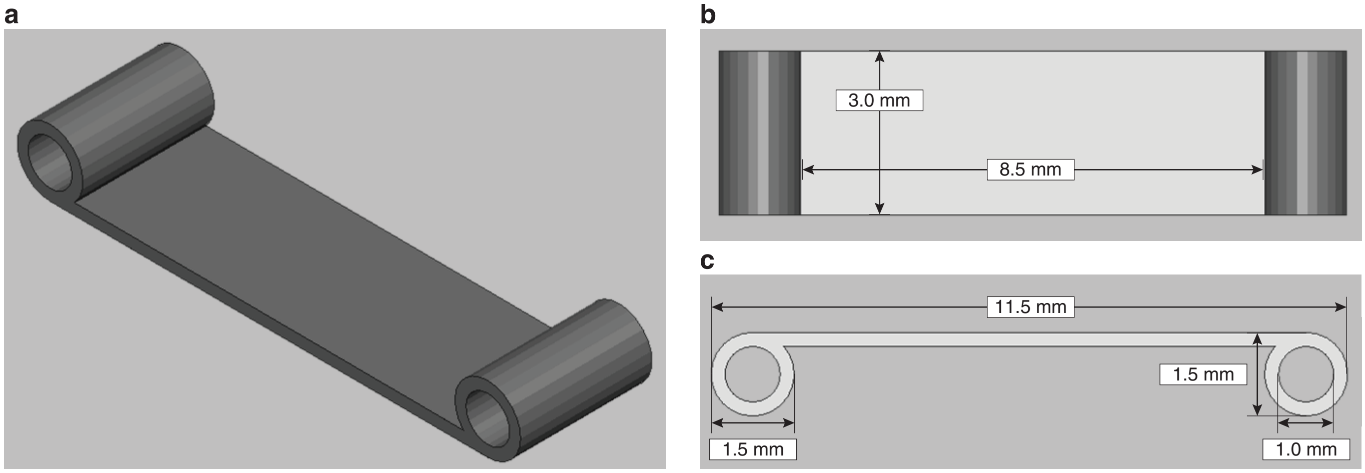

Supplementary Figure 1. 3D-render of the ferromagnetic scaffold for stabilized colon imaging. (**a**) Isometric view of the ferromagnetic scaffold design. Orthographic projection of the ferromagnetic scaffold design with dimensions: (**b**) top view and (**c**) front view.

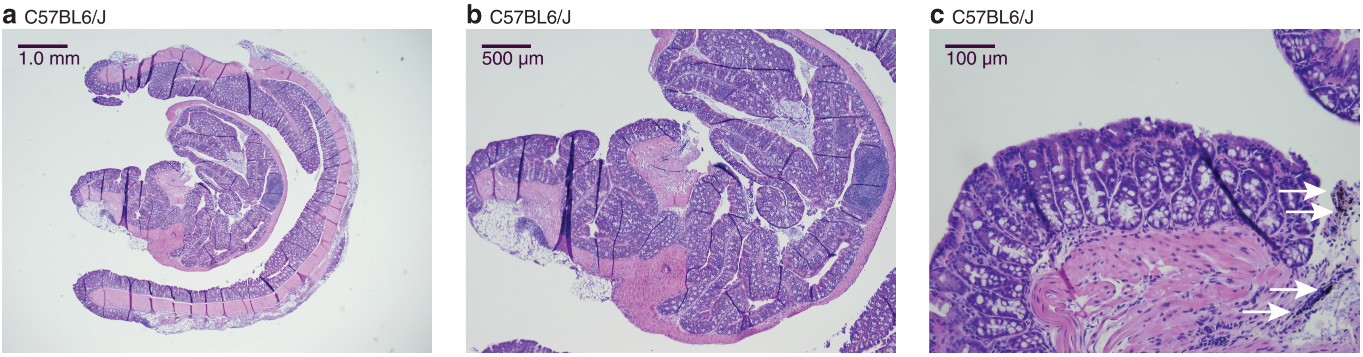

Supplementary Figure 2. Representative images of hematoxylin and eosin staining. Hematoxylin and eosin stained colon from a C57BL6/J mouse at (**a**) 20X, (**b**) 40X, and (**c**) 100X magnification. Arrows indicate visible tattoo regions in the tissue. All scale bars are as labeled.

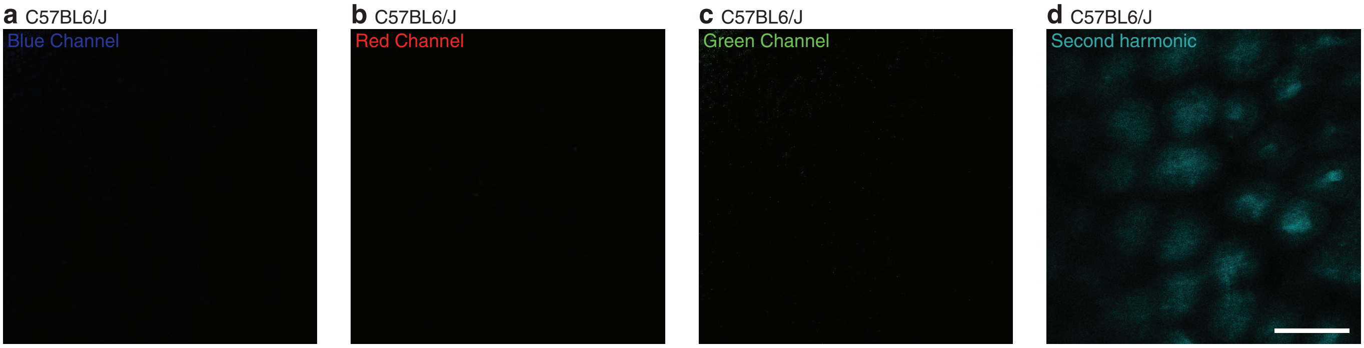

Supplementary Figure 3. Low autofluorescence in *in vivo* imaging in C57BL6/J mice. Representative images via the colonic window in C57BL6/J mice in the (**a**) blue, (**b**) red, and (**c**) green channels, as well as (**d**) the second harmonic generation. Scale bars is 100 μm.

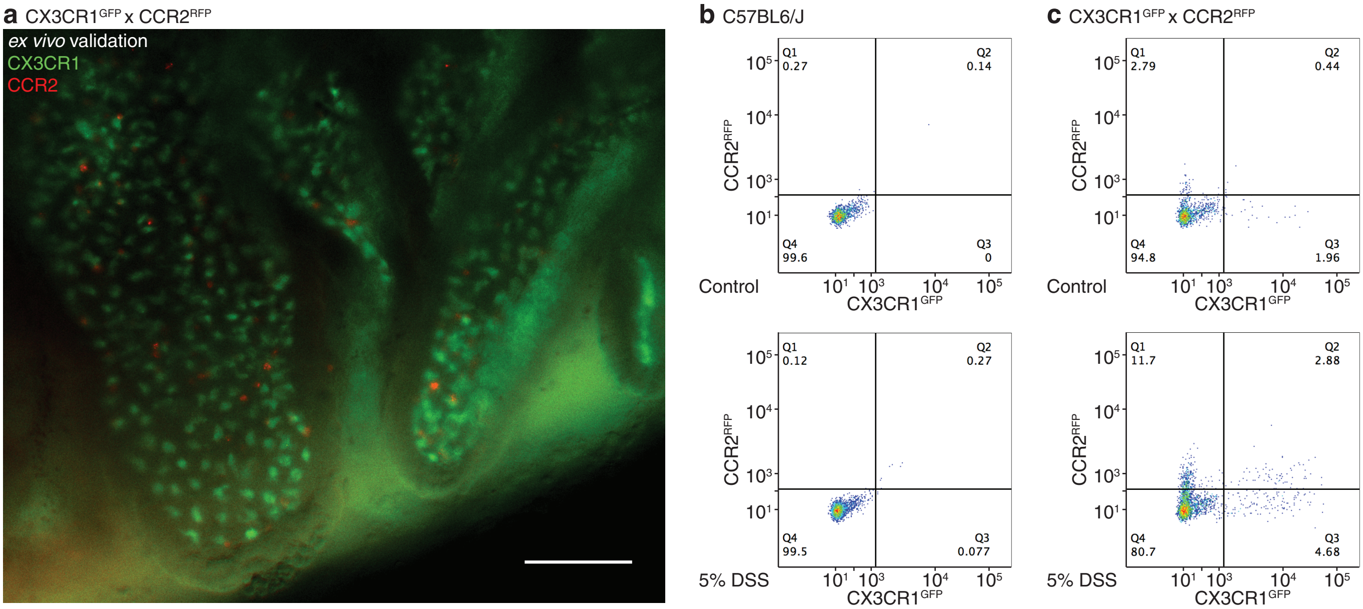

Supplementary Figure 4. *Ex vivo* CX3CR1^{GFP} x CCR2^{RFP} DSS inflammation model validation. (**a**) Representative image from CX3CR1^{GFP} x CCR2^{RFP} excised tissue via fluorescent microscopy showing innate dendritic cells and inactivated monocytes (green) and activated mononcytes (red). (**b,c**) Quantification of CX3CR1-positive cells and CCR2-positive cells via flow cytometry from a (b) C57BL6/J mouse, and from a (c) CX3CR1^{GFP} x CCR2^{RFP} mouse. Scale bar is 100 μm.

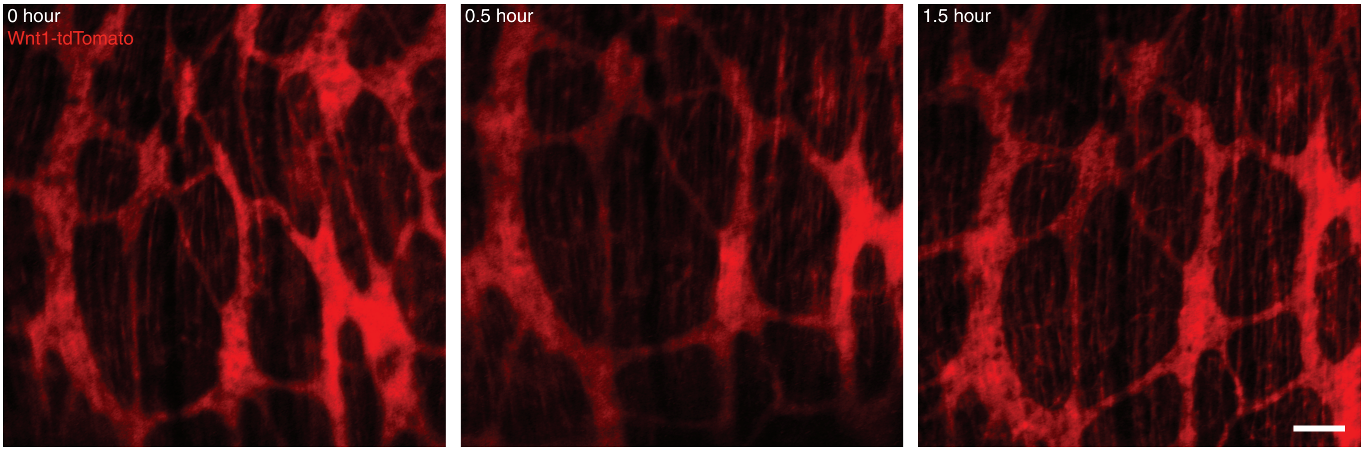

Supplementary Figure 5. Colonic myenteric plexus in a Wnt1-tdTomato mouse. Representative *in vivo* z-stack projections of the myenteric plexus through the colonic window. Images are captured at 0, 0.5, and 1.5 hours. Scale bar is 100 μm.

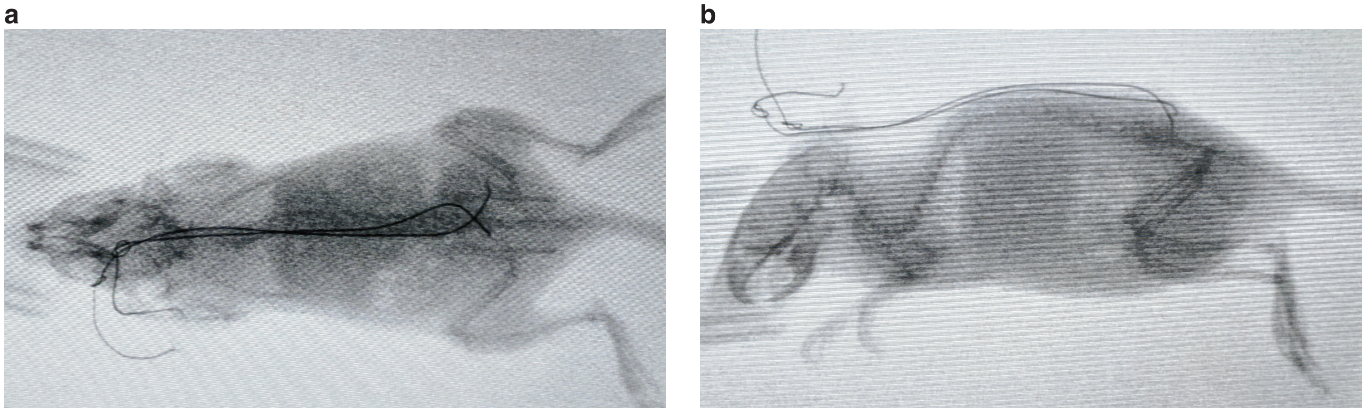

Supplementary Figure 6. X-ray fluoroscopy of a mouse with electrical stimulation leads. (**a**) Dorsal and (**b**) lateral view of a mouse with electrical stimulation leads implanted in the S2 intervebral foramen.

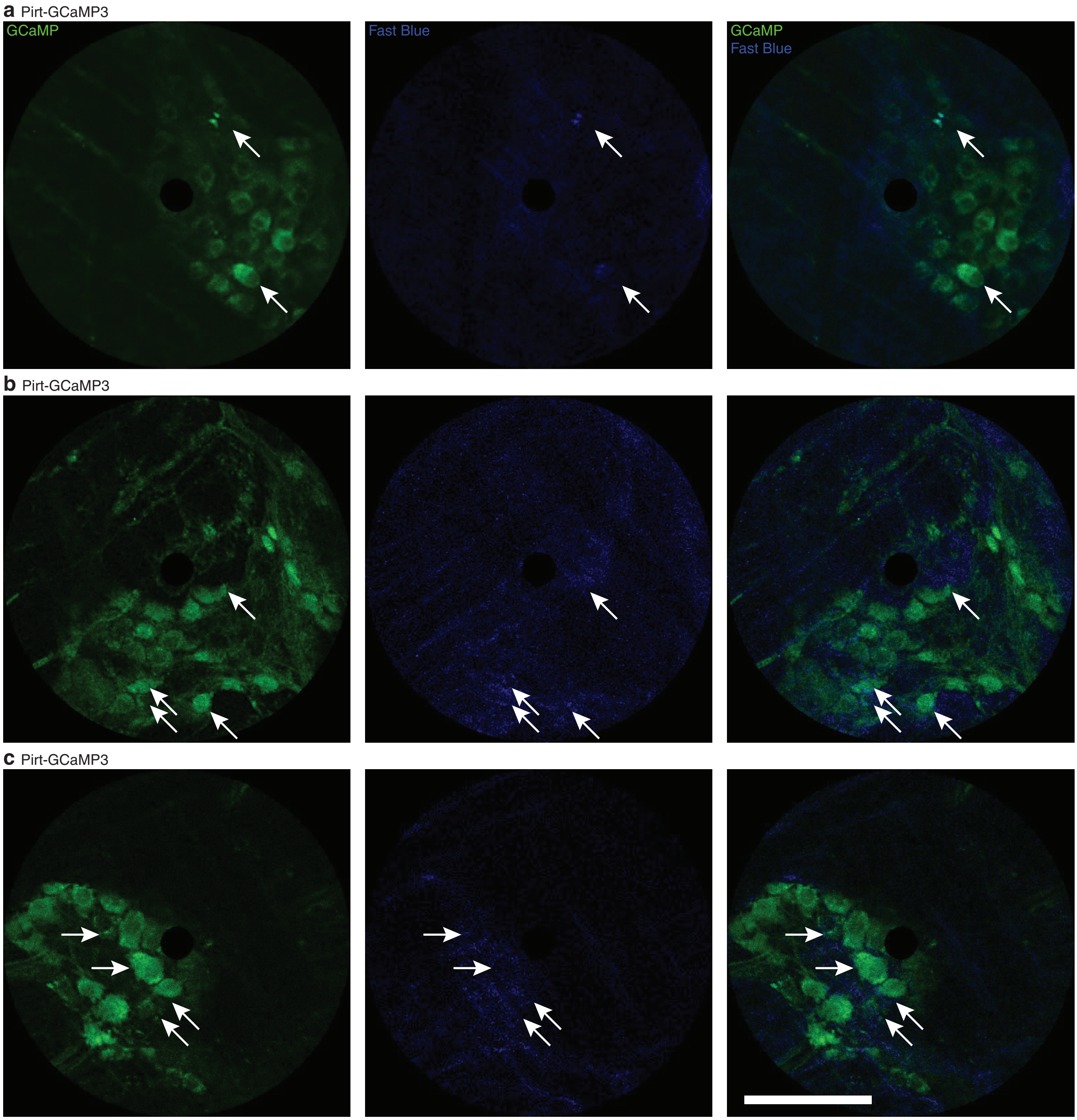

Supplementary Figure 7. Representative images of Fast Blue labeling in Pirt-GCaMP3 mice *in vivo*. (a,b,c) Representative images of GCaMP-positive cells (green) and cells labeled with Fast Blue (blue) in three mice. Arrows indicate double-positive cells. All scale bars are 100 μm.
