# Supplementary material for: An intravital window to image the colon in real time
Source: Nat Commun. 2019 Dec 11;10:5647. doi: 10.1038/s41467-019-13699-w (PMC6906443; doi:10.1038/s41467-019-13699-w)
Supplement: Supplementary file 3 — Description of Additional Supplementary Files [file 41467_2019_13699_MOESM3_ESM.pdf]

Description of Additional Supplementary Files

File name: Supplementary Movie 1

Description: Tissue movement in the colon is reduced by the ferromagnetic scaffold. Representative video via the colonic window from a C57BL6/J mouse with intravenous Texas Red-Dextran revealing colonic vasculature (red). (Left) Tissue movement with the ferromagnetic scaffold deactivated, and (right) with the ferromagnetic scaffold activated to restrain the colon against the window. Videos are from the same recording session in the same mouse, captured at 3.26 frames per second and played back at 40 frames per second. Scale bar is 100 μm.

File name: Supplementary Movie 2

Description: Mouse with colonic window moves unrestricted. Freely-behaving mouse after recovering from SNS electrode implantation and colonic window survival surgery.

File name: Supplementary Movie 3

Description: Cell alignment and video stabilization in post-processing. Representative video of colonic myenteric neurons (green) from a Pirt-GCaMP3 mouse along the processing pipeline: (a) unprocessed video, (b) sphere tracking in Imaris 8 with individual cell paths, (c) sphere tracking stabilization, and (d) the video post-processing, after stabilization in Fiji. Frames were captured at 0.8 frames per second and played back at 24 frames per second. All scale bars are 100 μm.

File name: Supplementary Movie 4

Description: Evoked motor response during SNS. Anesthetized C57BL6/J mouse exhibiting evoked motor response in the tail during SNS.

File name: Supplementary Movie 5

Description: Intravital calcium imaging with SNS. Representative video of colonic myenteric neurons (green) in a Pirt-GCaMP3 with SNS at 14 Hz delivered at 30 seconds. Frames were captured at 2.9 frames per second and played back at 40 frames per second. Scale bar is 50 μm.
