# Supplementary material for: An intravital window to image the colon in real time
Source: Nat Commun. 2019 Dec 11;10:5647. doi: 10.1038/s41467-019-13699-w (PMC6906443; doi:10.1038/s41467-019-13699-w)
Supplement: Supplementary file 9 — Reporting Summary [file 41467_2019_13699_MOESM9_ESM.pdf]

nature research

Corresponding author(s): Bradley B. Barth and Xiling Shen

Last updated by author(s): Oct 20, 2019

Reporting Summary

Nature Research wishes to improve the reproducibility of the work that we publish. This form provides structure for consistency and transparency in reporting. For further information on Nature Research policies, see Authors & Referees and the Editorial Policy Checklist .

Statistics

For all statistical analyses, confirm that the following items are present in the figure legend, table legend, main text, or Methods section.

n/a	Confirmed	
☐	☒	The exact sample size (n) for each experimental group/condition, given as a discrete number and unit of measurement
☐	☒	A statement on whether measurements were taken from distinct samples or whether the same sample was measured repeatedly
☐	☒	The statistical test(s) used AND whether they are one- or two-sided<br>*Only common tests should be described solely by name; describe more complex techniques in the Methods section.*
☐	☒	A description of all covariates tested
☐	☒	A description of any assumptions or corrections, such as tests of normality and adjustment for multiple comparisons
☐	☒	A full description of the statistical parameters including central tendency (e.g. means) or other basic estimates (e.g. regression coefficient) AND variation (e.g. standard deviation) or associated estimates of uncertainty (e.g. confidence intervals)
☐	☒	For null hypothesis testing, the test statistic (e.g. F, t, r) with confidence intervals, effect sizes, degrees of freedom and P value noted<br>*Give P values as exact values whenever suitable.*
☒	☐	For Bayesian analysis, information on the choice of priors and Markov chain Monte Carlo settings
☒	☐	For hierarchical and complex designs, identification of the appropriate level for tests and full reporting of outcomes
☒	☐	Estimates of effect sizes (e.g. Cohen's d, Pearson's r), indicating how they were calculated

Our web collection on statistics for biologists contains articles on many of the points above.

Software and code

Policy information about availability of computer code

Data collection	No software was utilized during data collection.
Data analysis	Data analysis was completed using a combination of commercially available software packages (JMP, Imaris, ImageJ) and custom code. Each program and application of it is clearly cited in the descriptions of data processing and analysis. Custom code is stored in an internal repository, and will be made available on request, as will any more specific details regarding parameters used by commercial software. We include the following code accessibility statement in our manuscript: The authors declare that the custom code used for data processing and analysis in this study are available from the corresponding author upon reasonable request. The data in Fig. 4 have associated custom code.

For manuscripts utilizing custom algorithms or software that are central to the research but not yet described in published literature, software must be made available to editors/reviewers. We strongly encourage code deposition in a community repository (e.g. GitHub). See the Nature Research guidelines for submitting code & software for further information.

Data

Policy information about availability of data

All manuscripts must include a data availability statement. This statement should provide the following information, where applicable:

- Accession codes, unique identifiers, or web links for publicly available datasets
- A list of figures that have associated raw data
- A description of any restrictions on data availability

The authors declare that the data supporting the findings of this study are available within the paper and its supplementary information files or available from the corresponding author upon reasonable request. The data in Fig. 1c,d,e,f; Fig. 2; Fig. 3; and Fig. 4 have associated raw data.

October 2018

Field-specific reporting

Please select the one below that is the best fit for your research. If you are not sure, read the appropriate sections before making your selection.

[x] Life sciences [ ] Behavioural & social sciences [ ] Ecological, evolutionary & environmental sciences

For a reference copy of the document with all sections, see nature.com/documents/nr-reporting-summary-flat.pdf

Life sciences study design

All studies must disclose on these points even when the disclosure is negative.

Sample size	Sample size was not pre-determined. As the primary goal of this publication is to describe a new tool rather than to explore novel biological phenomena, pilot studies to demonstrate the ability of the window to observe previously described phenomena typically involved 3-5 animals.
Data exclusions	Outlier data were only excluded when they could be linked to probable underlying problems in data collection, such as premature death due to extreme response to anesthetic, dislocation of stimulation leads, or gross internal physical abnormalities discovered during necropsy.
Replication	All data presented in this manuscript were successfully replicated internally and/or were consistent with previously published results. Individual data points representing unique animals are presented wherever data is quantified to support this statement.
Randomization	In most of our studies, animals served as their own controls. One of the primary values of the colon window is that animals can be observed at baseline during the preliminary portion of the study, and then re-measured throughout the course of treatment. Animals were randomly allocated into experiment groups where relevant to validate findings in traditional control/treatment experiments.
Blinding	Image processing and analysis was preformed blinded.

Reporting for specific materials, systems and methods

We require information from authors about some types of materials, experimental systems and methods used in many studies. Here, indicate whether each material, system or method listed is relevant to your study. If you are not sure if a list item applies to your research, read the appropriate section before selecting a response.

Materials & experimental systems

n/a	Involved in the study	
[x]	[ ]	Antibodies
[x]	[ ]	Eukaryotic cell lines
[x]	[ ]	Palaeontology
[ ]	[x]	Animals and other organisms
[x]	[ ]	Human research participants
[x]	[ ]	Clinical data

Methods

n/a	Involved in the study	
[x]	[ ]	ChIP-seq
[ ]	[x]	Flow cytometry
[x]	[ ]	MRI-based neuroimaging

Animals and other organisms

Policy information about studies involving animals; ARRIVE guidelines recommended for reporting animal research

Laboratory animals	Pirt-GCaMP3, Wnt1-cre:tdTomato, Lgr5-EGFP-IRES-creERT2, and CX3CR1-EGFPxCCR2-RFP mice were utilized. All animals were 6-12 weeks of age. Male and female animals were utilized.
Wild animals	This study did not utilize wild animals.
Field-collected samples	This study did not utilize field-collected samples.
Ethics oversight	All animal procedures were reviewed and approved by the Duke University Institutional Care and Use Committee (protocol A139-18-05 and 195-15-05) and Cornell University Institutional Care and Use Committee (protocol 2015-0029). They were conducted in accordance with the recommendations in the Guide for the Care and Use of Laboratory Animals84 and all relevant regulations for animal testing and research.

Note that full information on the approval of the study protocol must also be provided in the manuscript.

Flow Cytometry

Plots

Confirm that:

☒ The axis labels state the marker and fluorochrome used (e.g. CD4-FITC).

☒ The axis scales are clearly visible. Include numbers along axes only for bottom left plot of group (a 'group' is an analysis of identical markers).

☒ All plots are contour plots with outliers or pseudocolor plots.

☒ A numerical value for number of cells or percentage (with statistics) is provided.

Methodology

Sample preparation	CX3CR1GFP x CCR2RFP control mice or mice treated for 5 days with 5% DSS were euthanized after imaging. The colon was opened longitudinally, crypts were isolated, and then dissociated to single cells for sorting.
Instrument	BD DiVa
Software	FlowJo
Cell population abundance	We did not sort fractions or preform any analysis on sorted fractions; flow cytometry was only used as a validation of relative abundance of GFP+ and RFP+ populations observed during microscopy. Percent abundance of each gated quadrant is indicated in the figure.
Gating strategy	As single cells were not sorted or utilized in further experiments, FSC/SSC gating was not considered. Positive and negative gates were placed to delineate between clusters of positive and negative cells for the two native fluorophores expressed in our cells to produce broad quadrants. Gates are clearly drawn in the figure.

☒ Tick this box to confirm that a figure exemplifying the gating strategy is provided in the Supplementary Information.
